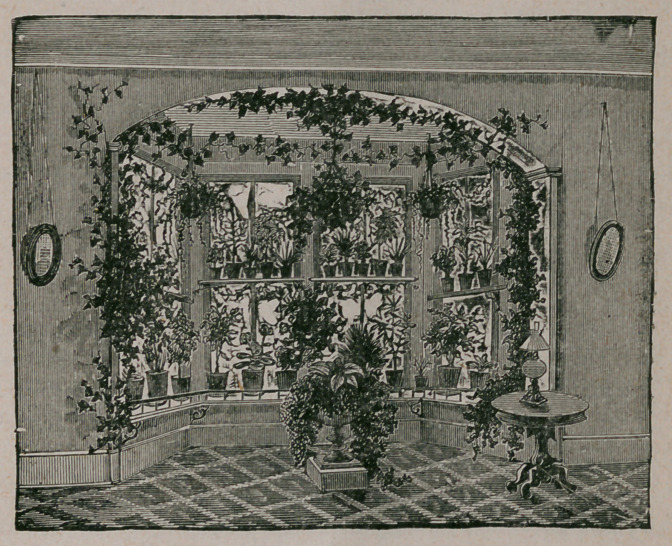# Household

**Published:** 1888-02

**Authors:** 


### HOUSEHOLD.

WINDOW GARDENING.—The articles on the above subject contained in our December and January numbers, were accompanied by illustrations of the manner of disposing flowers in ordinary windows, on a plane with the house walls. The above cut represents an ample bay window thus ornamented, in which the arrangement of flowers will be sufficiently understood without further comment.

THE ONION.—In spite of prejudices against it, the onion has always, by the great cooks of the day, been recognized as indispensable in cookery—equally as important in the humble Irish stew as in the high-class dishes of a Gouffe or a Francatelli. It is very true, as Swift wrote :

> "There is in every cook's opinion,
> No savory dish without an onion ;
> But, lest your kissing should be spoiled,
> The onion must be thoroughly boiled."

Although this distinctive flavor of the onion is lost in this long boiling, a richness and body is thus given to soups, sauces and stews obtainable in no other way. It would surprise many who declare that onions are intolerable from every point of view, to know how largely they enter into the composition of the dishes they most praise and enjoy. Although the properties of the onion are so valuable, they are far exceeded by those of garlic, which, although so largely used both as a condiment and a domestic medicine in the south of Europe and elsewhere, may not even be mentioned in England. We can only here refer to the use of garlic in cookery ; but it is so useful a thing that it would be well to accustom children very early to its moderate use, and to discourage the various prejudices against it. A great cook has said that "garlic should fly through the kitchen," thus indicating the very sparing way in which it should be employed to give that subtle and delicious flavor to many dishes so truly appreciated by the epicure.

The Use of Vinegar.—Experiments have shown that even so small a quantity of vinegar as one part in 5,000 appreciably diminishes the action of saliva upon starch. One part in 1,000 renders it very slow, and twice the latter quantity arrests it altogether. From this it is evident, says our contemporary, that vinegar, pickles, salads and other preparations in which vinegar is used are unwholesome, especially when taken with farinaceous food, such as bread and other preparations.

A Moth Preventative.—The following recipe for keeping moths out of clothing is a favorite in some families: Mix half a pint alcohol, the same quantity spirits turpentine and two ounces of camphor. Keep in a stone bottle and shake before using. The clothes or furs are to be wrapped in linen, and crumpled-up pieces of blotting paper, dipped in the liquid, are to be placed in a box with them, so that it smells strong. This requires renewing about once a year.

The Ages of Birds.—The following table is from an English source, and claims to be measurably correct as to the ages of the birds mentioned:

| | Years. | | Year. |
|---|---|---|---|
| Blackbird lives | 12 | Parrot lives | 6s |
| Blackcap | 15 | Partridge | 10 |
| Canary | 24 | Peacock | 25 |
| Crane | 24 | Pelican | 54 |
| Crow | 100 | Pheasant | 10 |
| Eagle | 100 | Pigeon | 25 |
| Fowl, common | 10 | Raven | 100 |
| Goldfinch | 15 | Robin | 10 |
| Goose | 50 | Skylark | 32 |
| Heron | 59 | Sparrow hawk | 40 |
| Lark | 13 | Swan | 100 |
| Linnet | 23 | Thrush | 10 |
| Nightingale | 18 | Wren | 3 |

—*Globe Democrat.*

Vines in the Room.—Few running plants are prettier for house decoration than those commonly known under the general name of Ivy. The German Ivy, Senecio scandens, is a rapid grower, but will not bear the cold as well as the English Ivy, which is the only real Ivy of the plants I am now noticing.

The English Ivy, if well treated, will live for many years. A friend of mine has one about fifteen years old, the largest one I ever saw. She keeps it on the piazza in summer; in the fall it is removed to the cellar. The Ivy is wound around two tall stalkes which are thrust into the soil in the keg which contains the plant. Water is given occasionally during the winter. If one has an English Ivy which seems to be dying, and its leaves wither and fade, they must not be picked off, but must be left to drop off. If the leaves are picked off when they show signs of decay, the tiny leaf-bud at the stem, so small as to be unnoticed, will be liable to be killed; but if let alone, a new leaf or shoot will come out of each one.

The Coliseum Ivy, Linaria cymbalarla, is beautiful when growing in a hanging pot at the window. It is easily raised from seed or cuttings.

The Ivy geraniums are very desirable for house plants, both on account of their leaves and their flowers.—*Vick's Magazine* for December.

## Figures and Tables

**Figure f1:**